# Acute Osteomyelitis of the Pubic Symphysis: A Case Report

**DOI:** 10.7759/cureus.40329

**Published:** 2023-06-12

**Authors:** Javier A Jara, Fernando A Inostroza, Felipe I Farias

**Affiliations:** 1 Orthopaedics and Trauma, Universidad de La Frontera, Temuco, CHL; 2 Orthopaedics and Trauma, Hospital Dr. Hernán Henríquez Aravena, Temuco, CHL; 3 Orthopaedics and Trauma, Hospital Dr. Hernán Henríquez Araven, Temuco, CHL; 4 Internal Medicine, Universidad de La Frontera, Temuco, CHL

**Keywords:** orthopedic sports medicine, mri, antibiotics therapy, pubic symphysis, osteomyelitis pubis

## Abstract

Pubis osteomyelitis is an uncommon disease, accounting for less than 1% of all bone infections. It occurs secondarily to hematogenous bacterial planting or direct inoculation. Clinically, it presents with intense acute pubic pain, limited mobility, and high fever, so it is rarely suspected initially. Its diagnosis can be easily confused with pubalgia, that do not respond to treatment. We present the case of a 17-year-old patient who sought consultation for three weeks of coxalgia associated with general discomfort and fever. Following a laboratory and imageological study, the diagnosis of acute pubis osteomyelitis was determined, which required surgical intervention and a subsequent pharmacological therapy for six weeks.

## Introduction

Acute osteomyelitis pubis (AOP) is a rare but potentially severe condition primarily affecting high-performance athletes and patients after pelvic surgery. It is estimated that it represents 1% of the total cases of osteomyelitis [1]. Clinical signs, such as symphysis pubis pain and fever, are the main manifestations [2]. The symptoms of AOP may be similar to those of osteitis pubis, so it is essential to make a correct assessment diagnosis to differentiate both conditions [3]. The diagnosis of AOP is based on clinical manifestations and imaging, with CT scans and MRI as the main methods of diagnosis [4,5]. The treatment of AOP is complex and must be approached in a multidisciplinary manner, which consists of a combination of IV antibiotic therapy and surgical debridement. Pharmacological treatment is based on IV broad-spectrum antibiotics that should include appropriate coverage for Staphylococcus aureus, Streptococcus, and Gram-negative bacteria. The minimum duration of antibiotic treatment is four to six weeks, and tissue removal is required in severe or refractory cases [6]. The prognosis of AOP depends on several factors, such as the time of diagnosis, the severity of the infection, and the response to treatment, with an average recovery time of 3-6 months [6]. A case of a 17-year-old patient who sought consultation for coxalgia that had been progressing for three weeks, associated with asthenia and fever, is presented. After the laboratory and imaging study, a diagnosis of AOP was determined, which required surgical intervention, followed by a six-week course of pharmacological therapy.

## Case presentation

Anamnesis

A 17-year-old male patient, who had been a soccer player since he was seven years old, presented to the ED. He had no history of morbid or surgical history, nor had he been in contact with individuals with tuberculosis, imprisoned, or used drugs. He reported a three-week history of intermittent fever reaching up to 40°C, associated with hypogastric pain that radiated to the left inguinal area. The symptoms were exacerbated during sports practice, repeatedly causing a need to stop physical activity. Since the onset of these symptoms, he reported a weight loss of approximately 5 kg, despite having no anorexia or associated gastrointestinal symptoms.

Physical exam

The patient was in generally stable condition, hemodynamically stable, eupneic, and afebrile, experiencing hypogastric pain rated 10/10 on the visual analog scale (VAS) without irradiation. The surgical team initially evaluated him and diagnosed a case of acute abdominal pain requiring surgery. Later, he was assessed by the traumatology team, with evidence of pain related to palpation at the pubic symphysis, without signs of skin infection or increased volume. There were no palpable adenopathies in the inguinal area without alterations to the active and passive mobilization of the lower extremities. The patient demonstrated overall M5 muscle strength with no associated neurological alterations. Orthopaedic tests, including the Roll test, FABER test, and FADIR test, were all negative.

Complementary exams

**Figure 1:**
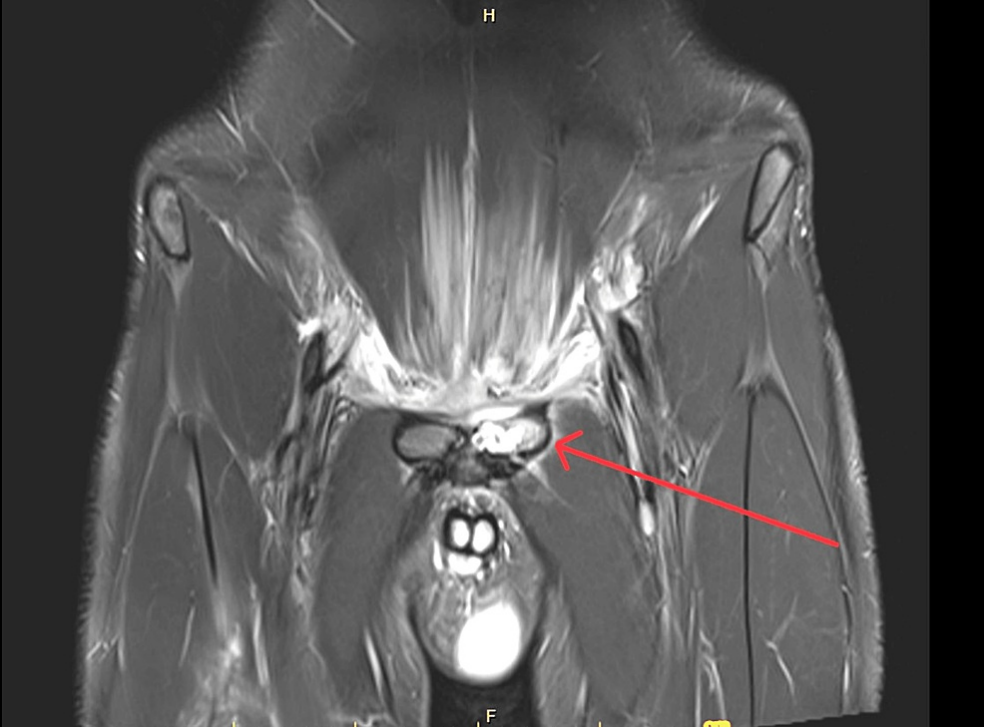
Magnetic nuclear resonance of pelvis (T2), coronal visualization. The arrow points to a lytic lesion on the upper margin of the left pubis. The lesion exhibits a high-to-intermediate T2 signal and shows significant enhancement with contrast medium.

**Figure 2:**
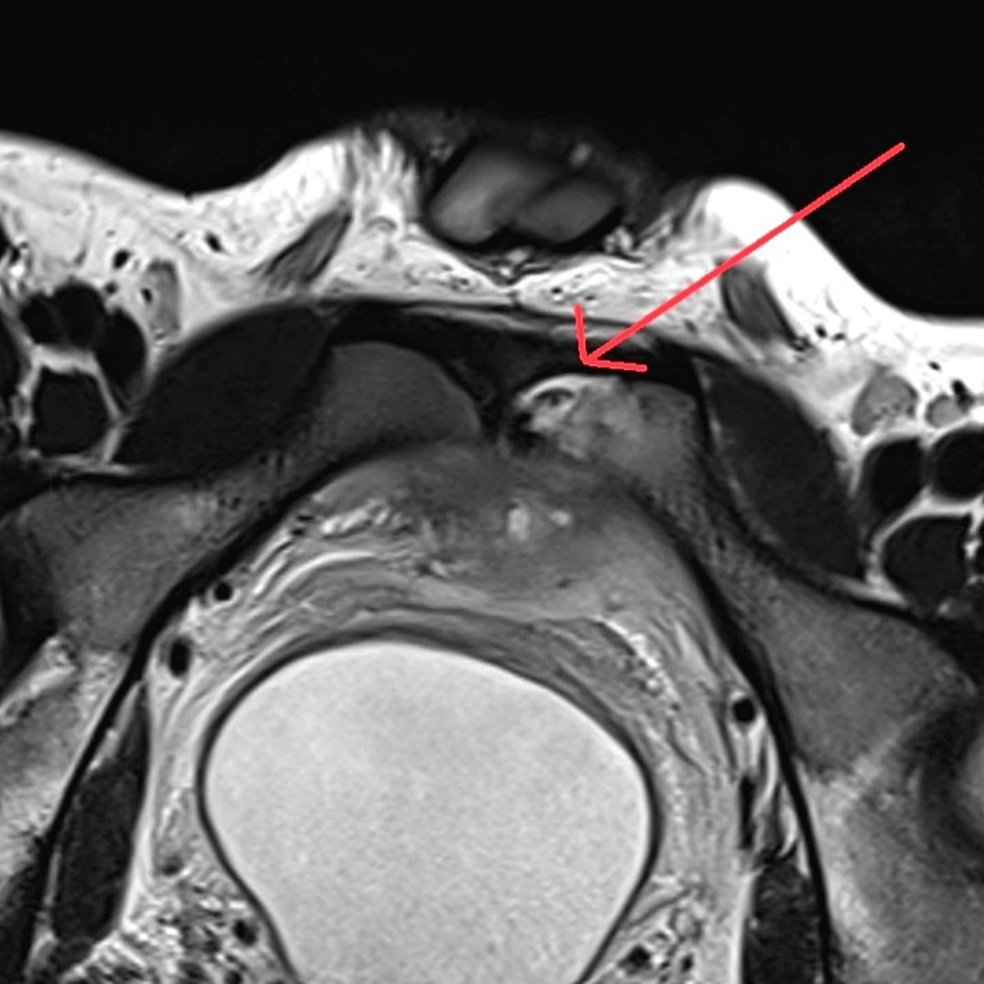
Magnetic nuclear resonance (T2): axial vision. The arrow indicates high-intensity injury compatible with lytic damage, accompanied by important inflammatory changes of perivesical adipose tissue.

In laboratory tests, the following results were notable: a C-reactive protein (CRP) level of 102 mg/L and leukocytosis of 12,000/mm3 with a predominance of polymorphonuclear cells (82%). Blood cultures were negative. Serological tests, including Venereal Disease Research Laboratory (VDRL), HIV, Hepatitis A Virus (HAV), Hepatitis B Virus (HBV), Hepatitis C Virus (HCV), and a test for Koch's bacillus, were all negative. The pelvis CT scan showed inflammatory changes associated with a lytic lesion in the anterior region of the left ileus pubic branch. The MRI showed a lytic lesion on the upper margin of the left pubis, with an intermediate high T2 signal, with significant enhancement with the contrast, measuring approximately 20 mm, accompanied by significant inflammatory changes of the perivesical adipose tissue and also with a small path that communicates with the right rectus abdominis forming a small intramuscular fluid collection of 10 x 6 mm, besides inflammatory changes in the posterior fascia of the left rectus abdominis (Figures 1-2).

Development

The patient entered the surgical ward, where cultures and biopsy of the pubic focus were taken by suprapubic access. In the intraoperative period, a whitish seropurulent discharge with increased consistency was observed at the pubic level. Three samples were taken, which resulted positive for multisensitive S. aureus. In the evaluation by the infectious disease team, the diagnosis of AOP was confirmed, so an IV cefazolin regimen was started for three weeks. During his hospitalization, the patient had favorable progress, persisting in good general condition, with painless walking and decreasing inflammatory parameters (CRP 5 mg/L and leukocytes 6,320/mm3). Hospital discharge was decided with an overlap to oral antibiotic with cotrimoxazole for six weeks, with a subsequent outpatient control with the traumatology and infectious disease teams. During the examination, he did not manifest pain during ambulation or physical activity. The intervention site exhibited no signs of recurrence or skin complications. Concerning laboratory tests, the CRP level was 2 mg/L, and the leukocyte count was 6,300/mm3. Due to satisfactory progress, a decision was made to medically discharge the patient (Table 1). The patient has been under follow-up for one year since hospital discharge and has been in good general health. He is asymptomatic and has not required further antibiotics.

**Table 1:** Results of laboratory tests during hospitalization. Table 1 shows changes in inflammatory parameters, and the impact of surgical intervention associated with pharmacological treatment during hospitalization.

	Initial	Three days post surgical cleaning	Fifth week post surgical cleaning	Ninth week post surgical cleaning
Creatinine (mg/dL)	0.71	0.97	0.95	0.75
Urea (mg/dL)	24.8	41.8	33	27.4
Ureic nitrogen (mg %)	11.6	19.5	15.4	12.8
Glomerular filtration rate (GFR) (ml/min/1.73m^2)	146.2	102	104.5	137.2
Sodium (mEq/L)	139	144	139	136
Potassium (mEq/L)	3.4	3.3	5	3.1
Chlorine (mEq/L)	104	105	103	99
Hemoglobin (g/dL)	12.5	13.8	15	15.1
Hematocrit (%)	38.1	41.7	45.2	45.5
Platelets (x10^3/uL)	326	397	286	385
Leukocytes (x10^3/uL)	12.85	6.99	8.82	11.02
C-Reactive Protein (CRP) (mg/L)	102	21	17	1.3

## Discussion

AOP is an infrequent pathology, defined as an inflammatory state caused by infectious microorganisms such as bacteria, mainly S. aureus, which coincides with the microorganism isolated in this case [7]. Other microorganisms such as group A and B streptococcus, pseudomonas, Streptococcus pneumoniae, and Mycobacterium tuberculosis are associated with AOP in patients who have undergone prior pelvic surgery or have immunosuppressive states such as diabetes mellitus [8,1]. The dissemination routes are hematogenous and contiguous to an infectious focus [1]. Among the risk factors, the following stand out pelvic surgeries, pelvic tumors, the use of IV drugs, and high-performance sports such as rugby, marathon races, or soccer. In the case under discussion, the patient is a young athlete who played soccer [2,8]. The clinical presentation is non-specific, characterized by pubic pain (5-13%), fever (74%), pain with hip movement (45%), and abdominal discomfort [8], so its incidence may be underdiagnosed, explaining the few reports currently available in the literature. Regarding diagnosis, the initial study includes laboratory tests, particularly inflammatory markers such as leukocytes and CRP, which provide indications of infectious processes; however, they have low specificity [8]. In terms of imaging studies, conventional radiography is not typically recommended for investigating AOP. This is because it can only show changes at the bone level 10-14 days after the onset of the infection, such as osteopenia, bone resorption, bone destruction, loss of cortical bone, and periosteal reaction. Thus, its true utility lies in ruling out other non-infectious pathologies [9]. CT scan is more sensitive than conventional radiography when assessing cortical characteristics, periosteal reaction, and the presence of air in adjacent soft tissues [10]. In this case, a lytic lesion is observed in the anterior region of the left iliopubic branch without the possibility of describing more characteristics.

Pelvic MRI is a highly sensitive tool for the early detection of AOP, and it can delineate the extent of cortical destruction, abnormalities in the bone marrow, and inflammation in the soft tissues [10]. This makes it possible to locate the specific lesion and the magnitude of its spread to areas such as the perivesical adipose tissue and adjacent musculature. Definitive diagnosis requires an etiological study based on cultures and a biopsy of the affected site [11]. In our case, a suprapubic approach was used, which allowed direct visualization of the affected bone tissue and, subsequently, the collection of samples of said tissue, increasing the efficiency and obtaining more representative cultures and biopsies. The treatment of choice consists of cleansing and debridement of the necrotic tissue, associated with an antibiotic plan adjusted to the results obtained in the cultures. In our case, IV cefazolin was initiated for three weeks, a period in which the patient remained hospitalized, with favorable progress, and later he completed six weeks with oral cotrimoxazole. As a result, his inflammatory parameters normalized, and he no longer showed any clinical symptoms. In literature, there is no established duration for antibiotic therapy; however, various case reports agree on continuing with antimicrobial management until the area with overflow tissue is replaced by vital tissue, and additionally, inflammatory parameters are within the normal range [12].

## Conclusions

AOP is an infrequent pathology that can occur in patients without apparent risk factors, so it should be suspected in all young athletes presenting with groin pain, fever, and a generally compromised condition. The suspicion can be further supported by elevated inflammatory parameters and imaging findings consistent with an acute inflammatory process.
